# Neuroprotective Effect of Quercetin during Cerebral Ischemic Injury Involves Regulation of Essential Elements, Transition Metals, Cu/Zn Ratio, and Antioxidant Activity

**DOI:** 10.3390/molecules26206128

**Published:** 2021-10-11

**Authors:** Ming-Cheng Lin, Chien-Chi Liu, Chin-Sheng Liao, Ju-Hai Ro

**Affiliations:** 1 Department of Medical Laboratory Science and Biotechnology, Central Taiwan University of Science and Technology, Taichung 406053, Taiwan; 2 Department of Nursing, National Taichung University of Science and Technology, Taichung 404336, Taiwan; vickyliu@nutc.edu.tw; 3 Laboratory Department, Chung-Kang Branch, Cheng-Ching General Hospital, Taichung 407211, Taiwan; p123765@yahoo.com.tw; 4 Department of Pharmacy, Chung-Kang Branch, Cheng-Ching Hospital, Taichung 407211, Taiwan

**Keywords:** quercetin, cerebral ischemia, essential element, transition metal, antioxidant

## Abstract

Cerebral ischemia results in increased oxidative stress in the affected brain. Accumulating evidence suggests that quercetin possesses anti-oxidant and anti-inflammatory properties. The essential elements magnesium (Mg), zinc (Zn), selenium (Se), and transition metal iron (Fe), copper (Cu), and antioxidants superoxide dismutase (SOD) and catalase (CAT) are required for brain functions. This study investigates whether the neuroprotective effects of quercetin on the ipsilateral brain cortex involve altered levels of essential trace metals, the Cu/Zn ratio, and antioxidant activity. Rats were intraperitoneally administered quercetin (20 mg/kg) once daily for 10 days before ischemic surgery. Cerebral ischemia was induced by ligation of the right middle cerebral artery and the right common carotid artery for 1 h. The ipsilateral brain cortex was homogenized and the supernatant was collected for biochemical analysis. Results show that rats pretreated with quercetin before ischemia significantly increased Mg, Zn, Se, SOD, and CAT levels, while the malondialdehyde, Fe, Cu, and the Cu/Zn ratio clearly decreased as compared to the untreated ligation subject. Taken together, our findings suggest that the mechanisms underlying the neuroprotective effects of quercetin during cerebral ischemic injury involve the modulation of essential elements, transition metals, Cu/Zn ratio, and antioxidant activity.

## 1. Introduction

Ischemic stroke, the most common type of stroke, often results in disability and death in older people. Ischemic stroke is characterized by increased oxidative stress due to reactive oxygen species (ROS) generation and antioxidant capacity depletion in the affected brain [1]. The generated ROS actively attack the polyunsaturated fatty acids via lipid peroxidation, resulting in oxidative brain damage and neuron death [1,2].

Quercetin is a plant flavonol that possesses polyphenol structure mainly found in vegetables and fruits such as red onion, cranberry, blueberry, and red apple [3]. Due to its bioactive polyphenol structure, quercetin exhibits anti-inflammatory and anti-oxidant properties [4]. Therefore, quercetin is widely used in botanical and traditional Chinese medicine to prevent tumors, cardiovascular diseases, and neurological diseases [3,4,5].

The essential elements magnesium (Mg), zinc (Zn), selenium (Se), iron (Fe), and copper (Cu) are required in multiple brain functions. Firstly, Mg is involved in energy metabolism, nerve cell function, anti-oxidation reactions, and anti-inflammatory responses [1,2,6]. Zn is responsible for brain development; wound healing; the synthesis of amino acids, proteins, and nucleotides; and is a component of superoxide dismutase [1,2]. In addition, the essential element Se is required for glutathione peroxidase activity and displays anti-inflammatory and anti-oxidant properties [1,7]. Fe is essential for the brain and is an integral part of hemoglobin and collagen [8,9,10]. Finally, Cu is involved in neurotransmitter biosynthesis, catalyzes electron transfer reactions in the mitochondria, and is a component of cytochrome-c oxidase [11,12]. It has been evidenced that disequilibrium in the concentrations of these essential elements is reported to be harmful to the brain [9,10,12].

Superoxide dismutase (SOD) and catalase (CAT) are antioxidant enzymes that protect the brain from ROS attack [13,14]. Depletion of either of these activities can attenuate the antioxidant capacity, resulting in increased oxidative stress and further brain injury [13,14]. The Cu/Zn ratio is a biomarker used to evaluate the status of inflammation, oxidative stress, and nutrition [15]. There are no studies discussing whether the neuroprotective mechanism of quercetin during cerebral ischemic injury is related to the regulations of essential elements and transition metals so far. Therefore, the main purpose of this study is to determine whether the neuroprotective mechanism of quercetin in the ipsilateral brain cortex involves the modulation of essential elements, transition metals, and antioxidant activity.

## 2. Results

### 2.1. Malondialdehyde (MDA) Levels in the Ipsilateral Brain Cortex Homogenates

**Figure 1:**
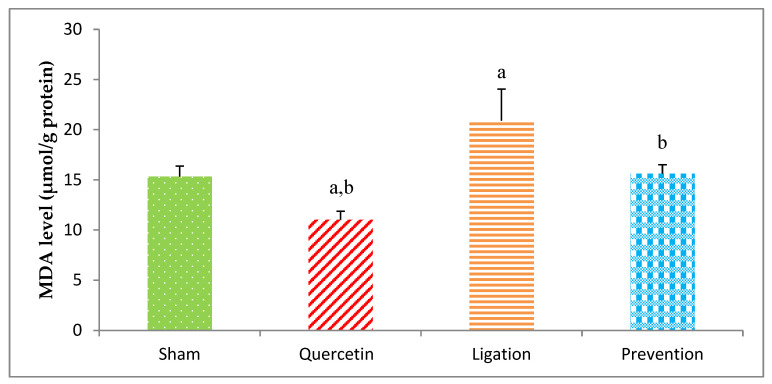
Malondialdehyde (MDA) concentration in the ipsilateral brain cortex homogenates. Data were expressed as mean ± S.D. The Kruskal–Wallis one-way analysis of variance (ANOVA) followed by Fisher’s Least Significant Difference test were used in this study. Difference of statistic was considered significant at *p* < 0.05. a: *p* < 0.05 vs. sham group; b: *p* < 0.05 vs. ligation group.

The end-product of lipid peroxidation is MDA, which is usually applied to evaluate oxidative injury. The value of the MDA in the group of sham, quercetin, ligation, and prevention was 15.34 ± 1.02, 11.02 ± 0.84, 20.89 ± 3.05, and 16.02 ± 1.08 μmol/g protein, respectively (Figure 1). Compared to the ligation group, the MDA concentrations were obviously low (*P* < 0.05) in the prevention group.

#### 2.1.1. Essential Trace Element Mg Levels in the Ipsilateral Brain Cortex Homogenates

**Figure 2:**
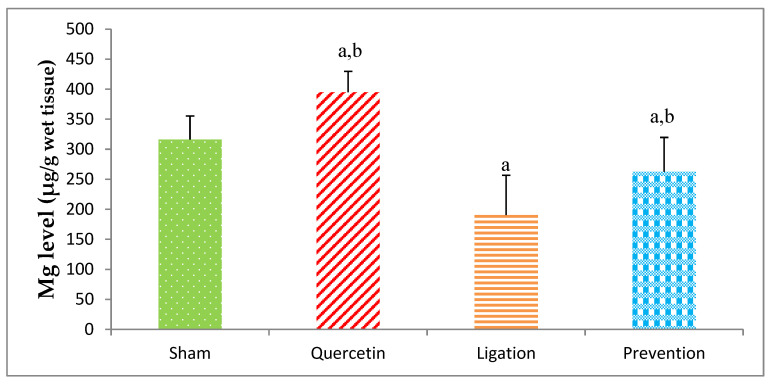
Magnesium (Mg) level in the ipsilateral brain cortex homogenates. Data were expressed as the mean ± S.D. The Kruskal–Wallis one-way analysis of variance (ANOVA) followed by Fisher’s Least Significant Difference test were used in this study. Difference of statistic was considered significant at *p* < 0.05. a: *p* < 0.05 vs. sham group; b: *p* < 0.05 vs. ligation group.

The Mg level in the sham, quercetin, ligation, and prevention groups was 316.04 ± 39.10, 394.71 ± 34.89, 190.58 ± 66.14, and 262.45 ± 57.28 μg/g, individually. A significant (*p* < 0.05) high Mg level was found in the prevention group as compared to the ligation group (Figure 2).

#### 2.1.2. Essential Trace Element Zn Levels in the Ipsilateral Brain Cortex Homogenates

**Figure 3:**
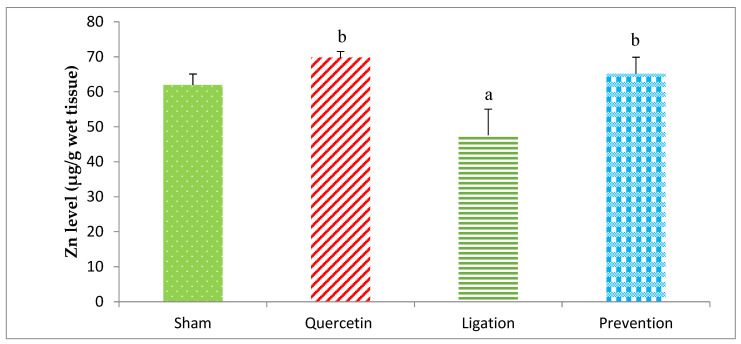
Zinc (Zn) level in the ipsilateral brain cortex homogenates. Data were expressed as the mean ± S.D. The Kruskal–Wallis one-way analysis of variance (ANOVA) followed by Fisher’s Least Significant Difference test were used in this study. Difference of statistic was considered significant at *p* < 0.05. a: *p* < 0.05 vs. sham group; b: *p* < 0.05 vs. ligation group.

The Zn concentration in the group of sham, quercetin, ligation, and prevention was 61.99 ± 3.06, 69.78 ± 1.72, 47.59 ± 7.42, and 65.15 ± 4.74 μg/g, respectively (Figure 3). The Zn concentration was obviously (*p* < 0.05) high in the prevention group relative to the ligation group.

#### 2.1.3. Essential Trace Element Se Levels in the Ipsilateral Brain Cortex Homogenates

**Figure 4:**
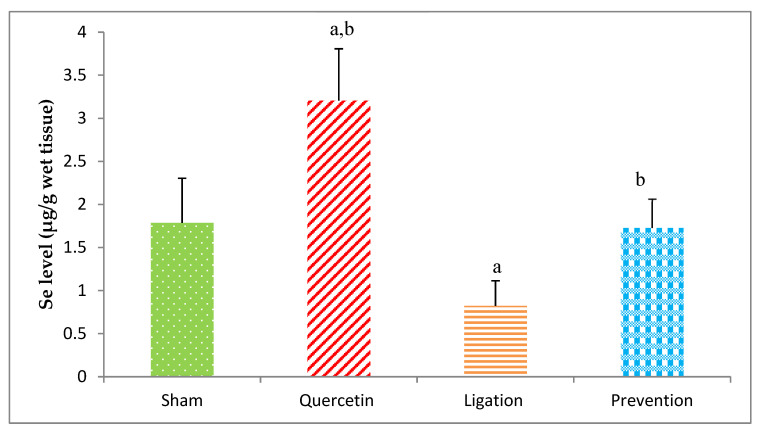
Selenium (Se) level in the ipsilateral brain cortex homogenates. Data were expressed as the mean ± S.D. The Kruskal–Wallis one-way analysis of variance (ANOVA) followed by Fisher’s Least Significant Difference test were used in this study. Difference of statistic was considered significant at *p* < 0.05. a: *p* < 0.05 vs. sham group; b: *p* < 0.05 vs. ligation group.

As listed in Figure 4, the Se level in the group of sham, quercetin, ligation, and the prevention was 1.78 ± 0.51, 3.20 ± 0.60, 0.820 ± 0.29, and 1.72 ± 0.33 μg/g, respectively. As compared to ligation group, the Se level was significantly elevated (*p* < 0.05) in the prevention group.

#### 2.1.4. Essential Trace Element Fe Levels in the Ipsilateral Brain Cortex Homogenates

**Figure 5:**
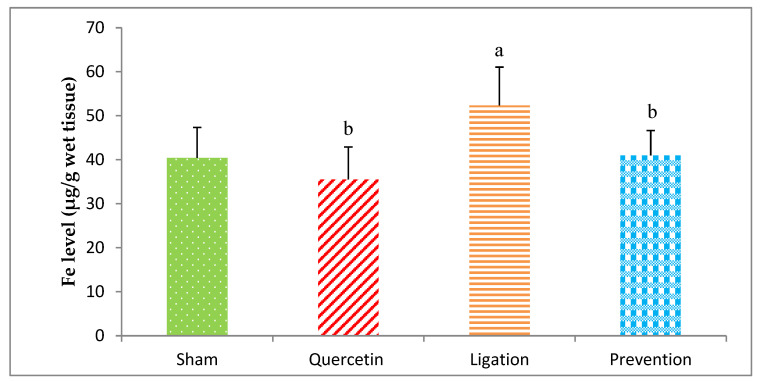
Iron (Fe) level in the ipsilateral brain cortex homogenates. Data were expressed as the mean ± S.D. The Kruskal–Wallis one-way analysis of variance (ANOVA) followed by Fisher’s Least Significant Difference test were used in this study. Difference of statistic was considered significant at *p* < 0.05. a: *p* < 0.05 vs. sham group; b: *p* < 0.05 vs. ligation group.

The Fe level in the group of sham, quercetin, ligation, and prevention was 40.43 ± 6.90, 35.54 ± 7.37, 52.34 ± 8.69, and 40.98 ± 5.67 μg/g, respectively. A statistical decrease (*p* < 0.05) of the Fe level was showed in the prevention group relative to the ligation group (Figure 5).

#### 2.1.5. Essential Trace Element Cu Levels in the Ipsilateral Brain Cortex Homogenates

**Figure 6:**
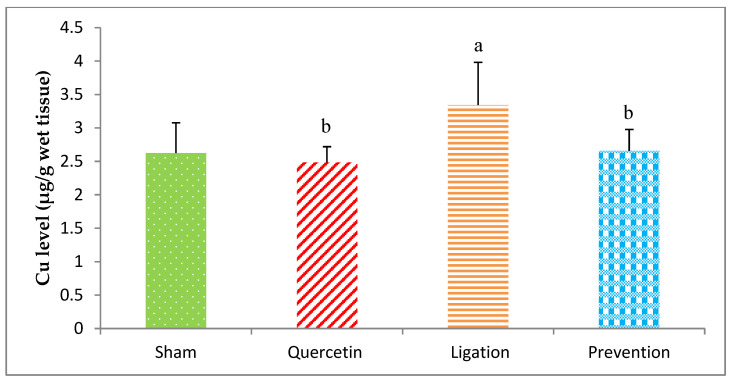
Copper (Cu) level in the ipsilateral brain cortex homogenates. Data were expressed as the mean ± S.D. The Kruskal–Wallis one-way analysis of variance (ANOVA) followed by Fisher’s Least Significant Difference test were used in this study. Difference of statistic was considered significant at *p* < 0.05. a: *p* < 0.05 vs. sham group; b: *p* < 0.05 vs. ligation group.

The Cu level in the group of sham, quercetin, ligation, and prevention was 2.62 ± 0.44, 2.48 ± 0.23, 3.34 ± 0.64, and 2.65 ± 0.32 μg/g, individually (Figure 6). Relative to the ligation group, the Cu level was markedly low (*p* < 0.05) in the prevention group.

### 2.2. Antioxidant Activity in the Ipsilateral Brain Cortex Homogenates

#### 2.2.1. The SOD Activity in the Ipsilateral Brain Cortex Homogenates

**Figure 7:**
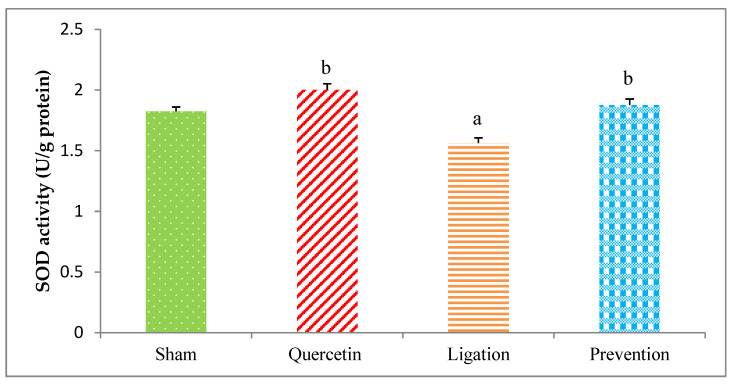
Superoxide dismutase (SOD) activity in the ipsilateral brain cortex homogenates. Data were expressed as the mean ± S.D. The Kruskal–Wallis one-way analysis of variance (ANOVA) followed by Fisher’s Least Significant Difference test were used in this study. Difference of statistic was considered significant at *p* < 0.05. a: *p* < 0.05 vs. sham group; b: *p* < 0.05 vs. ligation group.

The SOD value obtained from the group of sham, quercetin, ligation, and prevention was 1.82 ± 0.04, 2.00 ± 0.04, 1.56 ± 0.03, and 1.87 ± 0.05 U/g protein, respectively. As compared to the ligation group, the SOD activity was statistically higher (*P* < 0.05) in the prevention group (Figure 7).

#### 2.2.2. The CAT Activity in the Ipsilateral Brain Cortex Homogenates

**Figure 8:**
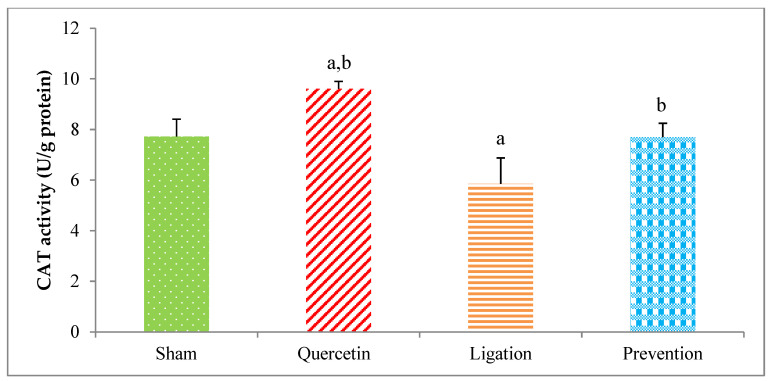
Catalase (CAT) activity in the ipsilateral brain cortex homogenates. Data were expressed as the mean ± S.D. The Kruskal–Wallis one-way analysis of variance (ANOVA) followed by Fisher’s Least Significant Difference test were used in this study. Difference of statistic was considered significant at *p* < 0.05. a: *p* < 0.05 vs. sham group; b: *p* < 0.05 vs. ligation subject.

The antioxidant of the CAT activity in the group of sham, quercetin, ligation, and prevention was 7.72 ± 0.68, 9.60 ± 0.29, 5.84 ± 1.02, and 7.69 ± 0.55 U/g protein, respectively (Figure 8). Relative to the ligation group, the CAT value was significantly increased in the prevention group (*p* < 0.05).

### 2.3. The Cu/Zn Ratio in the Ipsilateral Brain Cortex Homogenates

**Figure 9:**
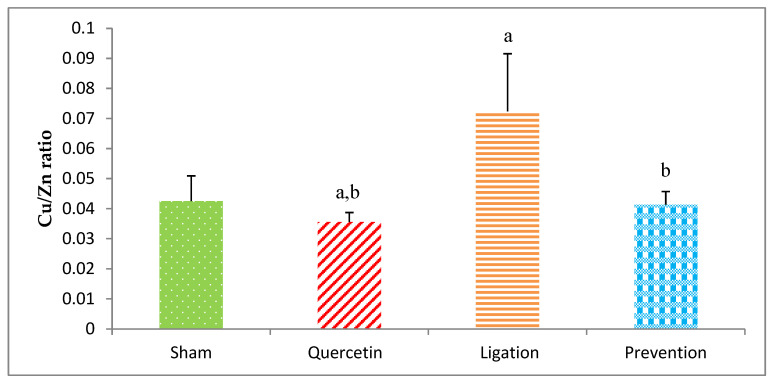
Cu/Zn ratio in the ipsilateral brain cortex homogenates. Data were expressed as the mean ± S.D. The Kruskal–Wallis one-way analysis of variance (ANOVA) followed by Fisher’s Least Significant Difference test were used in this study. Difference of statistic was considered significant at *p* < 0.05. a: *p* < 0.05 vs. sham group; b: *p* < 0.05 vs. ligation group.

The Cu/Zn ratio in the ipsilateral brain cortex homogenates in the group of sham, quercetin, ligation, and prevention was 0.043 ± 0.009, 0.036 ± 0.005, 0.072 ± 0.02, and 0.041 ± 0.005, respectively (Figure 9). As compared to the ligation group, the Cu/Zn ratio was significantly increased in the prevention group (*p* < 0.05).

## 3. Discussion

Our findings reveal that a disruption in the balance between essential element concentrations and depletion of antioxidant activity are indeed involved in the pathophysiology of acute ischemic stroke. Further, we observed the fact that the neuroprotective mechanism of quercetin during acute cerebral ischemic injury is associated with increasing the concentrations of the essential elements Mg, Zn, and Se and the antioxidant enzymes SOD and CAT and decreasing the concentrations of malondialdehyde (MDA), Fe, Cu, and the Cu/Zn ratio in the ipsilateral brain cortex.

The brain is particularly vulnerable to oxidative attack due to its relatively low levels of antioxidant activity and high composition of polyunsaturated fatty acids [1]. Ischemic stroke is caused by the interruption of blood flow to the affected brain. Under this condition, a lack of energy supplementation causes metabolic impairment and generates toxic ROS. Once the generated ROS level overwhelms the antioxidant capacity of the brain, ROS-mediated detrimental effects, including inflammation, calcium overload, excite-toxicity, protease activation, and mitochondrial dysfunction, cause neuronal cell injury or death [1,2]. This neuron loss results in partial body paralysis, cognitive impairment, and memory disorders in ischemic stroke patients.

As already mentioned, the polyphenol components of quercetin possess ROS scavenging ability [3]. The neuroprotective effects of quercetin against oxidative stress-mediated toxicity have been attributed to its anti-oxidant, anti-inflammatory, and anti-apoptotic activities and effects on signal transduction [3,4,5]. To date, no study has addressed whether the mechanism underlying the neuroprotective activity of quercetin during cerebral ischemia involves alteration of essential trace element concentrations in the affected brain. A previous study reports that quercetin acts against oxidative stress and necrosis via activation of an aspartate-specific cysteine protease that disrupts the association between RIPK1 and RIPK3 [16]. Quercetin also inactivates nuclear factor kappa B expression, resulting in decreased expression of the inflammatory cytokines tumor necrosis factor-α, cyclooxygenase-2, prostaglandin E synthase, and interleukin 1-beta [16,17,18]. A study in an animal model revealed that quercetin decreases adenine-induced chronic renal disease by attenuating lipid peroxidation and inflammation [19]. Furthermore, cadmium-induced neurotoxicity can be ameliorated by quercetin via decreased ROS and lipid peroxidation [20]. Together, these studies demonstrate that the protective effect of quercetin against ROS-mediated lipid peroxidation and neurotoxicity is related to its anti-oxidant and anti-inflammatory properties. In the present study, we observed that the ligation-induced increase in brain MDA levels was prevented by quercetin treatment before ischemic injury. This finding confirms that quercetin effectively reduces cerebral ischemia-mediated lipid peroxidation in the affected brain cortex through its anti-oxidant activity, consistent with results of the previous investigations.

Mounting attention has focused on the relationship between essential elements and human diseases [1,2,6]. Essential elements are vital to human health, as they are involved in a multitude of biochemical reactions in cells [2,6]. Because of their fundamental roles in the brain, many studies have investigated Mg, Zn, Se, Fe, and Cu [2,6,15,17]. Mg is the most abundant intracellular cation and exhibits a wide range of biological activities, including anti-oxidant functions, energy production, modulation of glucose transport across the cell membrane, calcium antagonist activity, and anti-inflammatory effects [6,21]. Our previous study suggested that neuroprotection by magnesium sulfate during cerebral ischemia occurs via attenuation of the infarct volume and glutamate level and maintenance of the glucose concentration [22]. One clinical study reported an inverse association between Mg levels and the outcome of post-stroke patients [21]. Other studies show that decreased Mg level is implicated in the occurrence of oxidative stress, inflammation, lipid peroxidation, metabolic syndrome, and cerebral ischemia [23,24]. Additionally, a clinical study has evidenced that patients with low serum Mg levels during the acute phase of ischemic stroke may be more susceptible to neurologic deterioration and worse outcomes, and the trend of our experimental finding is in line with the preceding study [25]. As mentioned above, it is notable that a decrease in Mg level is harmful to the brain. Here, we observed that cerebral ischemia in rats resulted in diminished Mg levels, but treatment with quercetin before ischemia markedly increased Mg levels. We speculate that quercetin greatly increases Mg levels during the cerebral ischemic insult; under this condition, elevated Mg is helpful not only to increase the energy supply to the ischemic brain cortex but also to reduce cerebral ischemia-mediated ROS levels, thereby attenuating lipid peroxidation and further oxidative brain injury. This study is the first to show that the neuroprotective effect of quercetin involves elevation of Mg levels in the ipsilateral brain cortex during cerebral ischemic injury.

Zn is involved in a variety of biological functions such as brain development, wound healing, anti-oxidation, and anti-inflammation [1,2,15]. An investigation showed that Zn exerts antioxidant effects to protect cells from ROS attack to prevent further deleterious lipid peroxidation [26]. Conversely, a recent study suggested that a marked decrease in serum Zn concentration can be found in patients with acute ischemic stroke [15]. Besides, decreased Zn levels are associated with cerebral ischemia-mediated oxidative stress and directly correlate with the progression of cellular injury [27]. Additionally, Zn deficiency down-regulates the expression and function of nuclear factor erythroid 2–related factor 2 (Nrf2) proteins and affects the status of intracellular heme oxygenase-1, SOD, and glutathione S-transferase [28]. Our present results show that cerebral ischemic injury in rats results in decreased Zn levels, but treatment with quercetin before ischemia clearly prevents this detrimental phenomenon. Quercetin effectively increases Zn levels in the ipsilateral brain cortex. This positive effect is useful for mitigating cerebral ischemia-mediated lipid peroxidation and further oxidative brain injury.

Essential trace element Se is important to human health because of its activity as an anti-oxidant, anti-inflammatory, promoter of enzyme activity, and ROS elimination [29]. Se deficiency results in a decreased antioxidant capacity and increased oxidative stress level [29,30]. A clinical study in northeastern Poland revealed that Se level is significantly decreased in patients with acute ischemic stroke [15]. Cisplatin-induced oxidative stress in the kidneys is attenuated by Se through decreasing ROS-mediated lipid peroxidation together with increasing the levels of antioxidant defense enzymes SOD and CAT [31]. One study in animals reports that patulin-induced oxidative brain injury can be mitigated via Se supplementation [32]. In addition, an ethanol-induced gastric mucosal lesion in rats is reduced by Se via attenuating lipid peroxidation and increasing SOD and CAT levels [33]. An in vivo study reports that treatment of Alzheimer’s disease model rats with sodium selenate clearly reduces the associated functional and neurodegenerative deficits [34]. Additionally, clinical evidence indicates that supplement of Se to lymphocytes from Alzheimer’s disease patients significantly decrease oxidative stress by reducing ROS, stimulating anti-aging genes, and elevating antioxidant activity [35]. A previous study also reports that treating animals with quercetin before ischemic brain injury resulted in markedly increased antioxidant activity of SOD, CAT, and GSH peroxidase in the hippocampal pyramidal neurons [36]. In this present study, pretreating rats with quercetin before ischemic injury not only significantly increased the Se level but also elevated the antioxidant activity of SOD and CAT in the ischemic brain cortex. This is the first study to show that the neuroprotective effect of quercetin is related to increase Se levels during cerebral ischemic insult. Accordingly, we propose that the mechanism underlying the neuroprotective effects of quercetin on ischemic brain cortex during cerebral ischemia involve its induction of increased levels of Se, SOD, and CAT. These alterations induced by quercetin plausibly mitigate cerebral ischemia-mediated lipid peroxidation and further oxidative brain injury.

The essential element Fe is required for cells because it is involved in a variety of cellular functions [37]. Our preceding study has suggested that excessive Fe is implicated in ROS generation via the Fenton reaction so as to be implicated with the etiology of stroke [8]. Cerebral ischemic insult can generate numerous ROS, including superoxide radicals, hydrogen peroxide, and hydroxyl radicals. These toxic ROS directly react with polyunsaturated fatty acids via lipid peroxidation and also attack cell membranes, proteins, and DNA, resulting in oxidative injury and cell death in the affected brain tissues. Cerebral ischemia-induced hydrogen peroxide simultaneously interacts with the Fe through the Fenton reaction to generate toxic hydroxyl radicals, resulting in further deleterious lipid peroxidation in the ischemic brain cortex [2,37,38,39]. Evidence from our previous study suggested that acute ischemic stroke results in Fe overload that increases lipid peroxidation in the ischemic brain [2]. Other studies have proposed that intracellular Fe plays a critical role in hydrogen peroxide-induced oxidative injury and DNA damage [38,39]. Our present findings are consistent with our previous study and suggest that cerebral ischemic injury resulting in Fe overload can be reduced by pretreatment with quercetin. A recent study showed that quercetin is a potent chelator of Fe due to its polyphenol structure [40], and another reported that quercetin chelates Fe, thereby influencing Fe absorption in the gastrointestinal tract [41,42]. Based on our findings, we conclude that quercetin chelates Fe to decrease the concentration of Fe, thereby ameliorating the Fe-mediated Fenton reaction and further lipid peroxidation. Thus, less oxidative brain injury occurred in the quercetin-treated than untreated rats.

Cu is essential for the catalytic activity of many brain proteins, including those involved in catecholamine biosynthesis, respiration, and antioxidation. However, Cu exhibits neurotoxicity when present in excess by promoting the generation of hydroxyl radicals via the Fenton reaction [43,44]. Several studies propose that high plasma Cu levels are associated with increased risk of ischemic stroke and Alzheimer’s and Wilson’s disease [43,44]. Our findings show that cerebral ischemic injury in rats leads to Cu overload that can be reversed by pretreatment with quercetin. Quercetin is reported to chelate Cu, thereby suppressing the generation of hydroxyl radicals through the Fenton reaction pathway [45]. Based on our findings, we propose that the neuroprotective effects of quercetin in the ischemic brain cortex occur through its reduction of Cu levels via chelation, thereby attenuating the Cu-mediated Fenton reaction and further deleterious lipid peroxidation.

**Figure 10:**
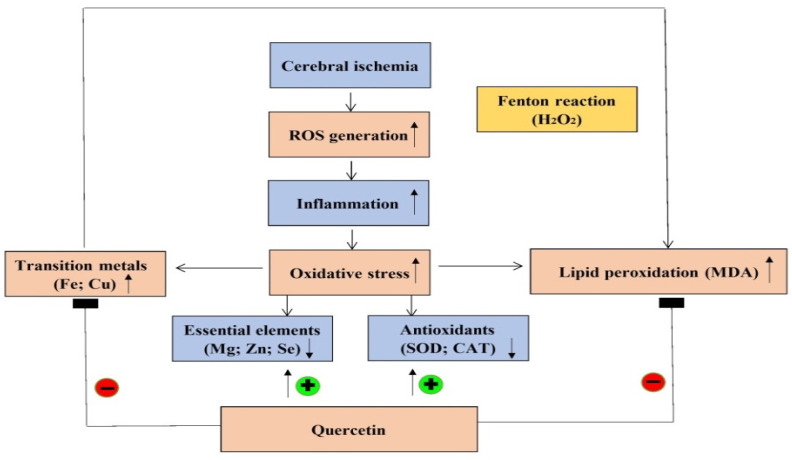
Mechanism underlying quercetin neuroprotection in the ipsilateral brain cortex.

The Cu/Zn ratio is used to evaluate the status of inflammation, nutrition, and oxidative stress [15]. Recent study evidences that a higher Cu/Zn ratio was observed in patients with larger brain infarct size and it is likely to become a useful marker of oxidative stress and inflammation in the pathogenesis of acute ischemic stroke [15]. On the other hand, a higher Cu/Zn ratio is often observed in age-related disorders such as neurodegenerative disease and systemic oxidative stress [15]. Additionally, several studies propose that acute ischemic stroke patients with larger brain infarct size exhibit a higher Cu/Zn ratio [46,47,48]. A clinical study from northeastern Poland suggests that a high serum Cu/Zn ratio directly correlates with acute ischemic stroke [15]. Another study reports a significant positive correlation between the inflammatory biomarker C-reactive protein and the Cu/Zn ratio [48]. Additionally, it is important to note that a high Cu/Zn ratio can be caused by increased Cu together with decreased Zn concentrations. While Zn possesses antioxidant ability, Cu initiates hydroxyl radical generation. Therefore, the Cu/Zn ratio is both a useful biomarker to predict the risk of ischemic stroke and an indicator of the degree of oxidative stress and inflammation in ischemic stroke patients. Our results are consistent with a previous study reporting that cerebral ischemic insults result in an elevated Cu but decreased Zn concentration, generating a high Cu/Zn ratio in the ipsilateral brain cortex. Pretreating rats with quercetin before ischemia effectively reverses this detrimental effect via reducing Cu but increasing Zn, resulting in the low Cu/Zn ratio observed in the present study. Importantly, this low Cu/Zn ratio indicates a beneficial effect of quercetin, showing that cerebral ischemia-induced oxidative stress and lipid peroxidation are attenuated, thereby mitigating further oxidative brain injury. In this present study, we used the stroke model by ligation of the right middle cerebral arterial (MCA) and the right common carotid arterial (CCA) for 1 h. Compared to other stroke models, this model has been suggested to produce the most reliable blood-brain barrier (BBB) disruption and cerebral infarction characterized by a shorter duration time of ischemia [49]. This is helpful for us to elucidate the early changes of the levels of essential elements, transition metals, antioxidant activity, and lipid peroxidation during cerebral ischemic injury. The justification for using the dose of 20 mg/Kg of quercetin in the present experiment is according to the previous study [50] in which the experimental conditions are similar to ours, such as using the male Sprague–Dawley rats, similar bodyweight of the experimental rats, as well as more significant and beneficial effects than 10 mg/kg in neuroprotection. The strength of this present study is that we first elucidate the mechanism underlying quercetin neuroprotection to the ischemic brain cortex during cerebral ischemic injury that is associated with the alterations of essential elements together with transition metals. On the other hand, the limitation of this study is the lack of the ipsilateral brain cortex samples so as to evaluate neuropathological changes of the ipsilateral brain cortex like infarct size/volume or neuronal death after cerebral ischemia. Overall, we summarize the mechanism underlying quercetin neuroprotection in the ipsilateral brain cortex during cerebral ischemic injury (Figure 10).

## 4. Materials and Methods

### 4.1. Animal Treatment and Samples Preparation

In this study, a total of forty Sprague–Dawley male rats with bodyweight ranging from 250 to 300 g were purchased from BioLASCO; the company of laboratory animal breeding and research in Taiwan. All rats were kept in stainless-steel mesh cages, housed under controlled conditions (22 ± 2 °C, 50 ± 20% relative humidity, 12-h light-dark cycle) with diet and water for 7 days for rats to adapt to the environmental conditions followed by randomly separated into four groups as below: sham (treated with physiological saline once in a day for consecutive 10 days); ligation (physiological saline was administered once in a day for consecutive 10 days before occlusion of the right common carotid artery (RCCA) and right middle cerebral artery (RMCA) for 60 min); quercetin (intraperitoneally injected rats with quercetin that is dissolved in physiological saline at a dosage of 20 mg/kg, which is purchased from Sigma-Aldrich, Merck, Germany, once in a day for consecutive 10 days); and prevention (pretreatment of rats with quercetin at a dosage of 20 mg/kg once in a day for consecutive 10 days and occlusion the artery of RCCA and RMCA for 60 min). All rats were anesthetized with chlorohydrate (400 mg/kg) intraperitoneally. On day 10, quercetin and saline were given 30 min prior to the RMCA plus the RCCA occlusion. The cerebral ischemic surgery was performed that right CCA, exposed through a ventral midline incision in the neck, was carefully isolated from the vago-sympathetic trunks and loosely encircled with sutures for further ligation. Following a midline incision, the skull was craniectomized to expose the right MCA. An 8–0 suture (blue monofilament polypropylene, DG, Davis-GECK, Wayne, N.J.) was positioned so that it encircled the MCA for further ligation. At the end of one-hour ligation, all rats were immediately sacrificed and the ipsilateral brain cortex tissue was collected, blotted, and rinsed with cold KCl (154 mM) solution and ready for further biochemical analysis. In this study, the animal use protocol listed and mentioned above has been approved by the Institutional Animal Care and Use Committee (IACUC) of Central Taiwan University of Science and Technology (107-CTUST-01).

### 4.2. Malondialdehyde (MDA) Concentration Analysis in the Ipsilateral Brain Cortex Homogenates

Experimentally, 0.2 g of the obtained ipsilateral brain cortex tissues was homogenized in the volume of 5 mL of ice KCl (154 mM) by Teflon pestles homogenizers, and the supernatants were immediately collected. For MDA analysis, 200 µL of the collected supernatant was mixed with 3 mL of the H_3_PO_4_ plus 800 µL of the KCl solution followed by being vortexed well. The standard solution of 1,1,3,3-tetra ethoxy propane was used to interact with the thiobarbituric acid (TBA) substance followed by boiling for 1 h. Finally, 4 mL of the butanol solution was added into the solution and vortexed well for 5 min followed by collection of the supernatant for MDA analysis. In brief, the detective principle of this present assay is based on the measurement of the pink color that is generated through the reaction of MDA with the TBA. The concentrations of the ipsilateral brain cortex MDA were assayed via spectrophotometer at the wavelength of 532 nm (U-1900, Hitachi, Japan).

### 4.3. Analysis of Enzyme Activity in the Ipsilateral Brain Cortex Homogenates

Antioxidant activity of SOD was measured based on the procedures of Cayman’s superoxide dismutase assay kit which was purchased from Cayman Chemical Company (catalog number 706002, Ann Arbor, MI, USA). Basically, xanthine oxidase can react with the hypoxanthine so as to generate the superoxide radical (O_2_^•−^). The superoxide radical interacted with the tetrazolium salt and the enzyme activity of SOD was measured via the instrument of spectrophotometry (Thermo Scientific Multiskan Spectrum, Ann Arbor, MI, USA). The SOD activity was expressed in terms of unit per gram of protein concentration. On the other hand, the CAT levels were assayed by a catalase commercial kit purchased from Cayman Chemical Company (catalog number 707002, Ann Arbor, MI, USA). In brief, this analytical procedure is that the methanol reacts with hydrogen peroxide under the catalyzation of the CAT enzyme so as to generate the formaldehyde. Finally, the chromogen of 4-amino-3-hydrazino-5-mercapto-1,2,4-triazole was reacted with the formaldehyde and the CAT levels were measured via spectrophotometer (Thermo Scientific Multiskan Spectrum, USA), and enzyme activities were expressed in terms of U per gram of protein concentration.

### 4.4. Measurement of Essential Trace Element in the Ipsilateral Brain Cortex Homogenates

To measure essential trace elements, 0.2 g of the harvested ipsilateral brain cortex sample was wet-digested completely with 4 mL of the nitric acid of ultra-pure-grade overnight followed by collecting the suspension for measuring the concentration of Mg, Zn, Se, Fe, and Cu. In order to avoid contamination, all containers used were completely soaked with 50% nitric acid, rinsed with ultrapure water followed by drying in an oven at 50 °C for later use. To enhance the analytical sensitivity for Se analysis, a specific hollow cathode lamp so-called super-lamp (Victoria, Braeside, Australia) was used in the present study. The standard solution of each analyzed element was dissolved in the concentration of 0.1 mol/L nitric acid solution purchased from Merck, Darmstadt, Germany. The instrument of the SavantAA Z graphite furnace atomic absorption spectrophotometer purchased from the company of GBC Scientific Equipment Pty Ltd. in Darmstadt, Australia, with the longitudinal Zeeman Effect background correction and PAL4000 auto-sampler system used experimentally.

### 4.5. Protein Concentration Analysis in the Ipsilateral Brain Cortex Homogenates

The commercial kit of BioChain protein assay (USA) was used in the present study. Basically, the principle of this protein assay kit was improved by the method of Coomassie Blue G. Experimentally, the reagent was reacted with the protein and produced a blue color complex and its color intensity is paralleled with protein concentration. The protein concentration was measured via spectrophotometry (Thermo Scientific Multiskan Spectrum, Ann Arbor, MI, USA) at the wavelength of 595 nm in the present work.

### 4.6. Data Analysis

The obtained data were expressed as mean ± S.D. The experimental value was analyzed by the Kruskal–Wallis one-way analysis of variance (ANOVA) method. Also, if the obtained data exhibit significant differences among groups, the Fisher’s Least Significant Difference (FLSD) method was used to compare each group. Once the P-value was less than 0.05, the statistical differences were considered significantly in the present work. a: *P* < 0.05, vs. sham; b: *P* < 0.05, vs. ligation.

## 5. Conclusions

To our knowledge, this is the first study to show that the neuroprotective effects exerted by quercetin during cerebral ischemic injury involve the modulation of essential elements and transition metal concentrations in the ipsilateral brain cortex tissue. However, determining the detailed mechanism underlying this effect will require further study.

## Sample Availability

Samples of the compounds are not available from the authors.
